# HIV-Tat regulates macrophage gene expression in the context of neuroAIDS

**DOI:** 10.1371/journal.pone.0179882

**Published:** 2017-06-22

**Authors:** Loreto Carvallo, Lillie Lopez, Jorge E. Fajardo, Matias Jaureguiberry-Bravo, Andras Fiser, Joan W. Berman

**Affiliations:** 1 Department of Pathology, Albert Einstein College of Medicine, Bronx, New York, United States of America; 2 Department of Systems and Computational Biology, Albert Einstein College of Medicine, Bronx, New York, United States of America; 3 Department of Microbiology and Immunology, Albert Einstein College of Medicine, Bronx, New York, United States of America; George Mason University, UNITED STATES

## Abstract

Despite the success of cART, greater than 50% of HIV infected people develop cognitive and motor deficits termed HIV-associated neurocognitive disorders (HAND). Macrophages are the major cell type infected in the CNS. Unlike for T cells, the virus does not kill macrophages and these long-lived cells may become HIV reservoirs in the brain. They produce cytokines/chemokines and viral proteins that promote inflammation and neuronal damage, playing a key role in HIV neuropathogenesis. HIV Tat is the transactivator of transcription that is essential for replication and transcriptional regulation of the virus and is the first protein to be produced after HIV infection. Even with successful cART, Tat is produced by infected cells. In this study we examined the role of the HIV Tat protein in the regulation of gene expression in human macrophages. Using THP-1 cells, a human monocyte/macrophage cell line, and their infection with lentivirus, we generated stable cell lines that express Tat-Flag. We performed ChIP-seq analysis of these cells and found 66 association sites of Tat in promoter or coding regions. Among these are C5, CRLF2/TSLPR, BDNF, and APBA1/Mint1, genes associated with inflammation/damage. We confirmed the association of Tat with these sequences by ChIP assay and expression of these genes in our THP-1 cell lines by qRT-PCR. We found that HIV Tat increased expression of C5, APBA1, and BDNF, and decreased CRLF2. The K50A Tat-mutation dysregulated expression of these genes without affecting the binding of the Tat complex to their gene sequences. Our data suggest that HIV Tat, produced by macrophage HIV reservoirs in the brain despite successful cART, contributes to neuropathogenesis in HIV-infected people.

## Introduction

Almost 80 million people worldwide have been infected with HIV since the beginning of the AIDS epidemic, and approximately 37 million individuals are now living with the virus [1]. As a result of the success of cART, infected people are living much longer. However, 40–70% of HIV infected people develop HIV-associated neurocognitive disorders (HAND), that are characterized by cognitive, motor and behavioral deficit [2–4].

HIV enters the CNS during the first weeks of peripheral infection, mainly through infected monocytes that cross the blood brain barrier (BBB) and infiltrate the brain parenchyma [5–7]. Within the CNS, monocytes differentiate into macrophages that produce virus, resulting in infection of other macrophages, microglia, and to a lesser extent astrocytes, as well as activation of these cells. These cells produce cytokines, chemokines, and viral proteins that continue to activate CNS cells, promote neuroinflammation, and often cause neuronal damage and apoptosis [8–14]. Macrophages are one of the major targets of HIV-1 in the CNS. HIV-infected macrophages persist within the brain for very long periods of time, where they serve as viral reservoirs. Even in the presence of successful cART, these viral reservoirs may still contain replicating virus, and even with complete suppression, continue to produce low levels of inflammatory factors and early viral proteins, including Tat, contributing to chronic neuroinflammation, neuronal damage, and the development of cognitive impairment [9, 15–23].

One of the viral proteins that is expressed by HIV-1 is Tat. Tat is the transactivator of transcription that is essential for transcriptional regulation and replication of the virus and is the first protein produced after HIV infection [24, 25]. Tat stimulates transcription of the HIV-1 genes. Tat also regulates gene expression in the host. Although Tat does not have DNA binding domains, many studies have shown that Tat mediates its effects on host gene expression through binding to host transcriptional regulator proteins, forming a Tat complex that includes RNA polymerase II, General Transcription Factors (TFIIB, TFIID, TFIIH, and TAF55), transcription regulators (p300/cAMP response element-binding protein (CREB), p300/CBP-associated factor (p/CAF), Tip60, human Nucleosome Assembly Protein-1 (hNAP-1), as well as the nucleosome remodeling complex SWI/SNF, and transcription factors (Sp1, NFAT, AP-1, NF-kB complex, p53, C/EBPb) [26–37].

The mechanisms by which Tat mediates gene expression in host cells are still not completely understood. In T cells, Tat binds to two phosphatase promoters, PTEN and Protein phosphatase 2, resulting in alteration of the Akt pathway, and inducing apoptosis [38]. Additionally, Tat regulates RNA polymerase II binding and release from DNA that control the initiation and elongation steps in several genes in T cells [37]. Tat also alters IFNAR2, CRLF1, and CASP8, that are related to apoptosis, in the U937 macrophage cell line [39]. Tat, together with amyloid beta, potentiated CCL2 and IL-6 promoter activity in human brain microvascular endothelial cells (HBMEC) [40]. In addition, Tat was shown to repress transcription of the mannose receptor, MHC class I, MHC class II, β2-microglobulin, and the BMP-2 receptor in U937 and in HeLa cells [41–45]. Much still needs to be understood about how Tat regulates gene expression in human macrophages. Despite the success of cART, it is not able to block the production of Tat, that continues to be transcribed in HIV-infected macrophages, even in the absence of viral replication. This can contribute to neurotoxicity and CNS damage [46, 47].

In this study, we characterized Tat mediated global gene expression in macrophages using ChIP-seq analysis. We identified 66 host genes with which Tat interacts in THP-1 cells that were generated to express Tat, and characterized the expression of 4 genes identified in this screening: C5, CRLF2, APBA1, and BDNF. We also demonstrated that a mutation of Tat, substitute lysine 50 for alanine, K50A, altered the expression of these genes, suggesting that lysine 50 of Tat is regulating C5, CRLF2, APBA1, and BDNF.

## Materials and methods

### Cell culture, generation, and selection of stable THP-1 cell lines

THP-1 cells (human monocytic cell line derived from a patient with acute monocytic leukemia) were a kind gift of Dr. Alejandro Villagra (The George Washington University). THP-1 cells were grown in RPMI supplemented with 10% fetal bovine serum, 1% penicillin-streptomycin, 1 mM sodium pyruvate, 10mM Hepes, 2 mM L-glutamine and 0.05 mM B-mercaptoethanol at 37°C in 5% CO2. To generate stable cell lines, THP-1 cells were infected with either a lentivirus that expresses Tat-flag [48] or luciferase (control) (Biosettia). The stable cells were then selected for their ability to grow in the presence of 1.5 ug/ml puromycin, a concentration determined previously by an antibiotic killing curve. Once generated, the THP-1-Tat-Flag and THP-1-luciferase cell lines were maintained in 1.5 ug/ml puromycin. All selected cell lines were evaluated for Tat-FLAG proteins by Western blot using mouse monoclonal anti-FLAG antibody (1:1000, F3165, Sigma). We performed an additional 4–5 independent sets of infections and selection of cell lines that are included in experiments.

### Cytoplasmic and nuclear extracts

Cytoplasmic and nuclear extracts from THP-1-Tat-Flag and THP-1-luciferase cell lines were prepared by the Dignam method [49, 50]. Cells were plated in 100-mm dishes at a density of 10x10^6^ and were induced to differentiate into macrophages by treatment with 30 ng/ml PMA for 24 hours. Cells were washed twice with ice-cold phosphate-buffered saline, and the entire isolation procedure was carried out on ice. Cells were gently resuspended in 250 ul of Homogenization buffer (10 mM HEPES at pH 7.2, 3 mM MgCl_2_, 10 mM KCl, 3 mM NaCl, 1 mM EDTA, 1 mM EGTA (pH 7.5), 0.5 mM PMSF, 1 μg/ml leupeptin, 1 μg/ml aprotinin, and 2 mM Na orthovanadate (pH 7.6) and incubated on ice for 20 minutes, lysed with 12.5 μl of 10% NP-40, and centrifuged at 10,000 g for 10 minutes at 4°C. The supernatant was collected and used as the cytoplasmic extract. The nuclear pellet was resuspended in 60 μl of nuclear extraction buffer (20 mM HEPES, pH 7.2, 1 mM MgCl_2_, 800 mM KCl, 25% glycerol, 0.5 mM EDTA, 1% NP-40, 0.5 mM PMSF, 1 μg/ml leupeptin, 1 μg/ml aprotinin, 2 mM Na orthovanadate, pH 7.6), agitated for 30 minutes at 4°C, and the nuclear debris was removed by centrifugation at 10,000 *g* for 15 minutes. The supernatant (nuclear extract) was collected and quick frozen in liquid nitrogen and stored at -80°C until use. Protein concentration was determined by Bradford assay, and both the cytoplasmic and nuclear proteins were subjected to Western blotting using mouse monoclonal anti-Flag antibody (1:1000, F3165, Sigma), rabbit anti-GAPDH (1:1000, #2118, Cell Signaling), and rabbit polyclonal anti-Pol II (Santa Cruz Biotechnology, 1:500, sc-899).

### Chromatin inmunoprecipitation (ChIP) and ChIP-seq assay

Chromatin immunoprecipitation was performed using 10x10^6^ cells as described [51] with modifications. All steps were performed at 4°C. THP-1-Tat-Flag and THP-1-luciferase cell lines (100-mm diameter plates) were differentiated with 30 ng/ml PMA for regular ChIP, and 100 ng/ml for ChIP-seq for 24 hours, and then were incubated for 15 minutes with 1% formaldehyde with gentle agitation. The cross-linking was stopped by addition of 0.125 M glycine and incubation for 10 minutes with agitation. Cells were then washed with PBS, scraped into 1 ml of PBS, and collected by centrifugation at 1,000g for 5 minutes. The cell pellet was resuspended in 1 ml of lysis buffer (50 mM Hepes pH 7.8, 20 mM KCl, 3 mM MgCl2, 0.1% NP-40, and a cocktail of protease inhibitors) and incubated for 15 minutes on ice. Cells were lysed by using a Dounce Homogenizer with 20 strokes. Cell extract was then collected by centrifugation at 1,000g for 5 minutes, and resuspended in 300 ul of sonication buffer (50 mM Hepes pH 7.9, 140 mM NaCl, 1 mM EDTA, 1% Triton X-100, 0.1% SDS, and a cocktail of protease inhibitors). To reduce the length of the chromatin fragments to approximately 200–500 bp (confirmed by electrophoretic analysis), the extract was sonicated with a Bioruptor (Diagenode) at the high setting. Cells were sonicated for a total of 30 cycles, 30 seconds on/30 seconds off. The sonicated mixture was centrifugated at 16,000g for 15 minutes at 4°C, and chromatin was collected from the supernatant. Cross-linked extracts (100 ul) were resuspended in sonication buffer to a final volume of 250 μl. Samples were precleared by incubation with normal mouse IgG plus protein A/G–agarose (Santa Cruz Biotechnology) for 2 hours at 4°C with agitation. After centrifugation at 1,000g for 5 minutes, the supernatant was collected and immunoprecipitated with 2 ug of anti-Flag antibody (F3165, Sigma, or #14793, Cell Signaling) overnight at 4°C with shaking. The immunocomplexes were recovered with the addition of 50 μl of protein A-agarose beads with incubation for 2 hours at 4°C with shaking. The complexes were washed twice with sonication buffer, once with sonication buffer plus 500 mM NaCl, once with LiCl buffer (100 mM Tris-HCl pH 8.0, 500 mM LiCl, 1.0% NP-40, and 1.0% deoxycholic acid), and once with TE buffer, incubating the solution at each washing for 5 minutes at 4°C with agitation. The protein-DNA complexes were then eluted by incubation with 100 μl of elution buffer (50 mM NaHCO3 and 1% SDS) for 15 minutes at 65°C. After centrifugation at 1,000g for 5 minutes, the supernatant was collected and incubated with 10 μg RNase A/ml for 1 hour at 42°C. NaCl was added to the mixture to a final concentration of 200 mM and incubated overnight at 65°C to reverse the crosslinking. The proteins were then digested with 200 μg/ml of proteinase K for 2 hours at 50°C. DNA was recovered using column DNA purification kit (Qiagen) and eluted with 50 μl of elution buffer. The primers used to amplify the sequence of the genes found by ChIP-seq are indicated in S1 Table. Precipitated DNA and input were then quantified by real-time PCR, using a StepOne Plus Real Time PCR system (Life technologies), and the Absolute Blue QPCR SYBR Low Rox Mix (Life technologies). Single product amplification was confirmed by melting curve analysis, and primer efficiency was close to 100% in all experiments performed. Quantification is expressed in arbitrary units indicating fold change over control (Luc), and target sequence levels were normalized to the input using the method of Pfaffl [52].

For ChIP-seq, \the libraries were prepared by the Epigenomic Shared Facility (Epigenomic Shared Facility of the Albert Einstein College of Medicine, NY). Libraries were multiplexed and single-end sequenced on the Illumina HiSeq 2000 machine with 100-bp read length.

### ChIP-seq data analysis

The single-end 100-nucleotide reads were aligned to the human genome (hg19 build) using gsna [53] and peak-calling was done with MACS [54]. The genomic locations of peaks with a False Discovery Rate (FDR) of 0.1 or smaller, as assigned by MACS, were collected for all samples and compared. Two peaks were defined to be common if their genomic locations showed any overlap. Peaks common to all three experimental samples and absent from control were selected, and their locations matched with the annotated start of genes in Refseq, as extracted from the refGene.txt file (*http://hgdownload.cse.ucsc.edu/goldenPath/hg19/database/refGene.txt.gz*). Peaks lying within the gene and 1000-nucleotide region immediately upstream of the annotated start of a first exon, referred to as the promoter region, were kept for further analysis. Sequences of the regions that contain selected peaks were obtained with the fastaFromBed program of the Bedtools package [55]. Peaks were visualized using the Integrative Genomics Viewer (http://www.broadinstitute.org/igv/).

### Quantitative real-time PCR

THP-1-Tat-Flag and THP-1-luciferase cell lines were differentiated to macrophages with PMA (30 ng/ml) for 24 and 48 hours. Total RNA was extracted using TRIzol reagent (Life Techonologies) and cDNA obtained with the SuperScript VILO (Life Techonologies). Target mRNA was quantified using a StepOne Plus Real Time PCR system (Life technologies) and Taqman Gene expression master mix (Life technologies). Probes used for PCR amplification were: 18S (Hs99999901_s1, Life technologies), C5 (Hs00156197_m1, Life technologies), CRLF2 (Hs00845692_m1, Life technologies), APBA1 (Hs00154104_m1, Life technologies), BDNF (Hs02718934_s1, Life technologies), Col6A6 (Hs01029204_m1, Life technologies), NCAN (Hs00189270_m1, Life technologies), with the cycling parameters of 2 minutes at 50°C, 10 minutes at 95°C, 15 seconds at 95°C, and 60 seconds at 60°C for 40 cycles. Quantification is expressed in arbitrary units, relative to THP-1-luciferase that was set to 1, and target mRNA levels were normalized to 18S expression using the method of Pfaffl [52].

### Mutant construction

To generate K50ATat-Flag mutant, site-directed mutagenesis was performed by using the QuikChange II XL Site-Directed Mutagenesis kit (Agilent Technologies) with Tat-Flag as a template DNA. The mutation was confirmed by DNA sequencing (Genomics Facility of the Albert Einstein College of Medicine, NY).

### Statistical analysis

All statistical analyses were performed with GraphPad Prism 6. Wilcoxon signed-rank tests were performed. Error bars represent the median ± S.D.; *P < 0.05, **P < 0.01, ***P < 0.001. For comparison between TatFlag and K50ATatFlag gene expression, Mann-Whitney tests were performed. Error bars represent the median ± S.D.; ^#^P < 0.05, ^##^P < 0.01, ^###^P < 0.001, ^####^P < 0.0001.

## Results

### Generation of stable THP-1 cell lines expressing Tat-Flag

**Fig 1:**
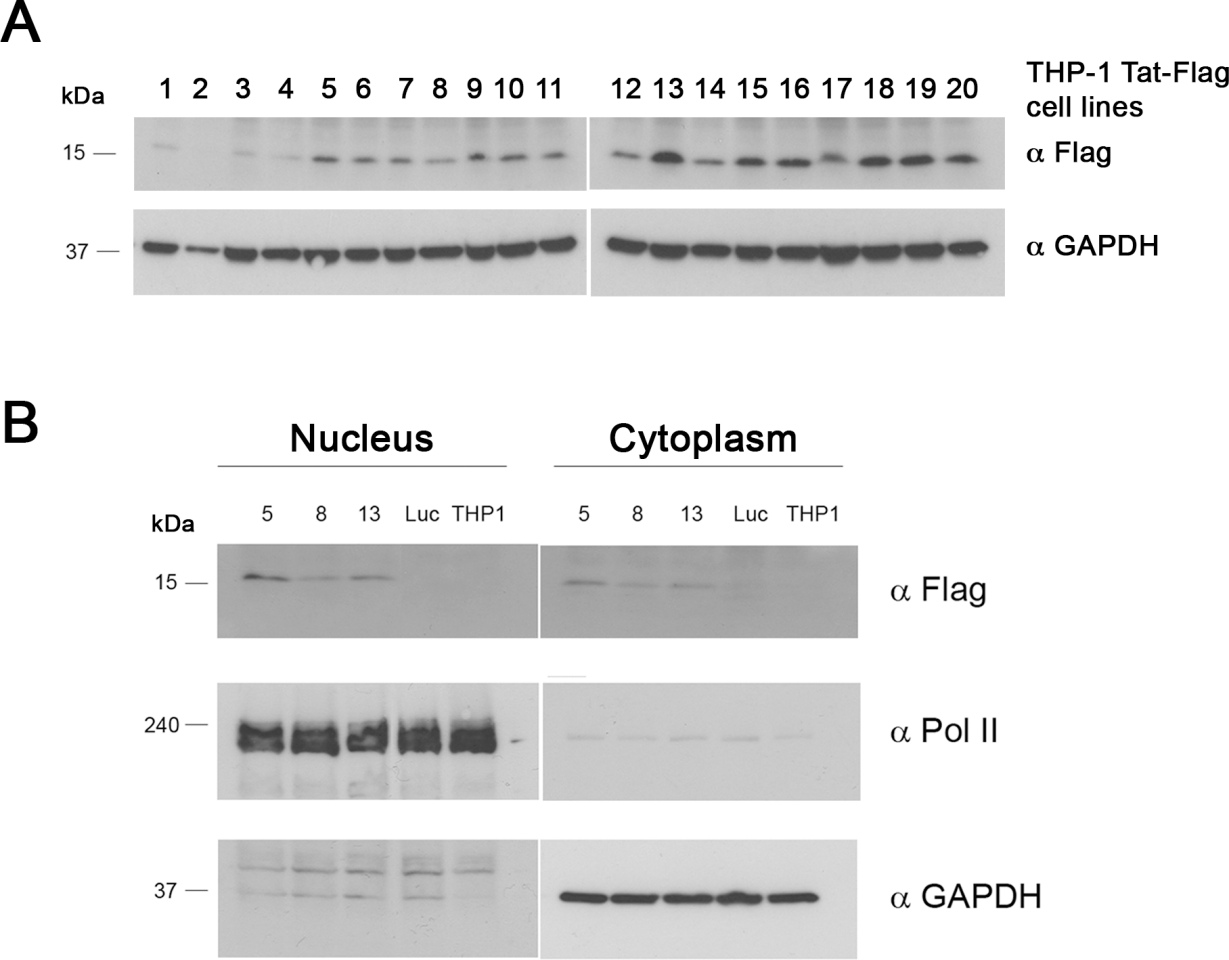
Generation of stable THP-1 cell lines that express Tat-Flag or Luciferase. We infected THP-1 cells with a lentivirus to generate stable cells that express Tat-Flag or luciferase. **(A)** THP-1 cells infected with Tat-Flag lentivirus and selected with puromycin (1.5 ug/ml) for 6 days. The cells were diluted to obtain stable cell lines. Protein lysates were analyzed by western blotting using anti-flag and anti-GAPDH antibodies. **(B)** THP-1-Tat-Flag and THP-1- luciferase cell lines were induced to differentiate into macrophages by treatment with PMA (30 ng/ml) for 24 hours. THP-1-Tat-Flag (cells lines 5, 8, and 13) and THP-1-Luciferase (Luc) as well as uninfected THP-1 cell were evaluated for nuclear and cytoplasmic expression of Tat-Flag after cell fractionation by Western bloting using anti-Flag, anti-Pol II (nuclear expression) and anti-GAPDH (cytoplasmic expression) antibodies. Almost all stable cells expressed Tat-Flag, and it was localized in the nucleus and cytoplasm. We obtained the same results with 4–5 independent sets of infections and selection of THP-1-Tat-Flag and THP-1-Luciferase cell lines.

To examine the role of Tat in macrophages by identifying and characterizing the genes with which it interacts, we generated stable THP-1 cell lines using lentivirus that express Tat-Flag. We used Tat-Flag instead of Tat by itself because the available antibodies to Tat are not highly specific. Therefore, we used antibody to Flag to recognize the expression of Tat because of its specificity and lack of background [38]. Additionally, it has been shown that the FLAG does not cause changes in gene expression by itself [37, 38, 56, 57]. THP-1 cells were infected with different multiplicities of infection (MOI, 1–50) of lentivirus, and were then selected for their ability to grow in the presence of 1.5 ug/ml puromycin. We also generated stable THP-1 cell lines infected with a lentivirus containing luciferase (Luc) as a negative control. Lysates of the THP-1 cell lines were prepared and analyzed by Western blotting with anti-Flag antibody. Blots were then stripped and reprobed with anti-GAPDH antibody. Most of the stable cell lines expressed Tat-Flag (Fig 1A). We chose 3 different lines for our experiments (cell lines number 5, 8, and 13, Fig 1A). THP-1-Tat-Flag and THP-1-luciferase cell lines were induced to differentiate into macrophages by treatment with PMA for 24 hours, and we performed subcellular fractionation and evaluated the nuclear and cytoplasmic expression of Tat-Flag by western blotting using an anti-Flag antibody, Pol II antibody (nuclear fraction) and GAPDH antibody (cytoplasmic fraction) Fig 1B). Only transduced cells expressed Tat-Flag.

### Global gene expression of differentiated THP-1 cells expressing Tat-Flag

**Fig 2:**
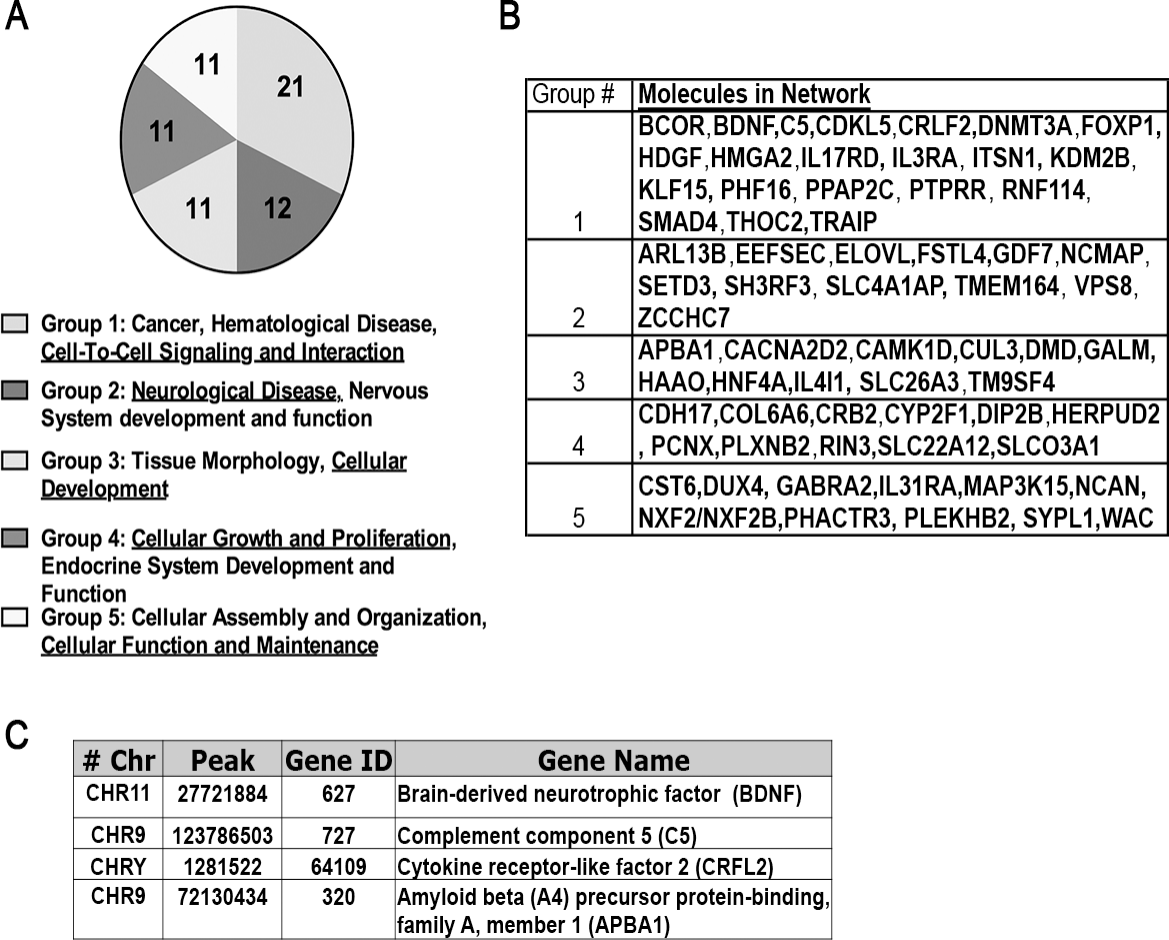
ChIP-seq analysis of PMA-differentiated THP-1 macrophage cells and IPA analysis. THP-1-Tat-Flag cell lines 5, 8, and 13, and THP-1-luciferase cell lines were treated for 24 hours with PMA and Chromatin Immunoprecipitation was performed. Cell lysates were prepared, precleared, and then immunoprecipitated with anti-Flag antibody. The DNA associated with Tat-Flag was recovered and libraries were generated for Illumina ChIP-Seq. The libraries were massively parallel sequenced, and analyzed with RefSeq, using hg19 human reference genome (see Methods). **(A)** Ingenuity Pathway Analysis (IPA) of the 66 genes identified by ChIP-seq analysis. We found 21 genes in Group 1, Cancer, Hematological disease, Cell-To-Cell Signaling and Interaction; 12 genes in Group 2, Neurological Disease, Nervous System development and function; 11 genes in Group 3, Tissue Morphology, Cellular Development; 11 genes in Group 4, Cellular Growth and proliferation, endocrine system Development and Function; and 11 genes in Group 5, Cellular Assembly and Organization, Cellular Function and Maintenance. **(B)** List of genes found by ChIP-seq classified in the five groups found by IPA. **(C)** Four genes (C5, CRLF2, APBA1, and BDNF) from the 66 genes associated with the Tat-Flag complex identified by ChIP-seq analysis, that have a potential role in inflammation, chemotaxis, and neuronal function were selected for subsequent study. Columns indicate the chromosome number, start of the peak, gene ID, and gene name.

To examine the host genes with which the Tat complex interacts in macrophages, we treated the THP-1 cell lines, THP-1-Tat-Flag (5,8 and 13), and the THP-1-luciferase for 24 hours with PMA to induce the cells to differentiate into macrophages, and ChIP assays were performed [51] using an anti-Flag antibody. DNA was recovered after the ChIP assay, and libraries for Illumina ChIP-Seq were generated. These libraries were massively parallel sequenced and analyzed using MACS (Model-based Analysis of ChIP-Seq), with a false discovery rate (FDR) of 0.1%. We obtained 1482, 1474, 949 and 136 peaks for the THP-1-Tat-Flag cell lines (5,8, and 13), and THP-1-luciferase cell line, respectively. The chromosomal locations of all of these peaks were compared, and 149 common to all of the samples were selected. Of these, 66 peaks were found to be either within a gene or in the 1000-nucleotide region upstream of the annotated start of a Refseq transcript (promoter). We visualized the peaks of the specific Tat-Flag binding site sequences using Integrative Genomics Viewer (IGV). We performed Ingenuity Pathway Analysis (IPA) of the 66 genes associated with the Tat-Flag complex identified by ChIP-seq analysis (Fig 2A), and classified the genes into 5 groups. These are: Group 1, Cancer, Hematological disease, Cell-To-Cell Signaling and Interaction, Group 2, Neurological Disease, Nervous System development and function, Group 3, Tissue Morphology, Cellular Development, Group 4, Cellular Growth and proliferation, endocrine system Development and Function, and Group 5, Cellular Assembly and Organization, Cellular Function and Maintenance. Fig 2B shows the genes found by ChIP-seq classified according to their functions. Among the genes identified by IPA are some shown to have a potential role in inflammation and, we hypothesized, neuroAIDS, including, complement component 5, C5, [58, 59], cytokine receptor-like factor 2/thymic stromal lymphopoietin, CRLF2/TSLPR, [60, 61], Amyloid Beta (A4) Precursor Protein-Binding, Family A, Member 1/ Munc18-1-interacting protein 1, APBA1/Mint1 [62], and Brain-derived neurotrophic factor, BDNF, [63–65]. We therefore chose to study these genes first (Fig 2C).

### Validation of ChIP-seq results, and regulation by Tat of candidate gene expression

**Fig 3:**
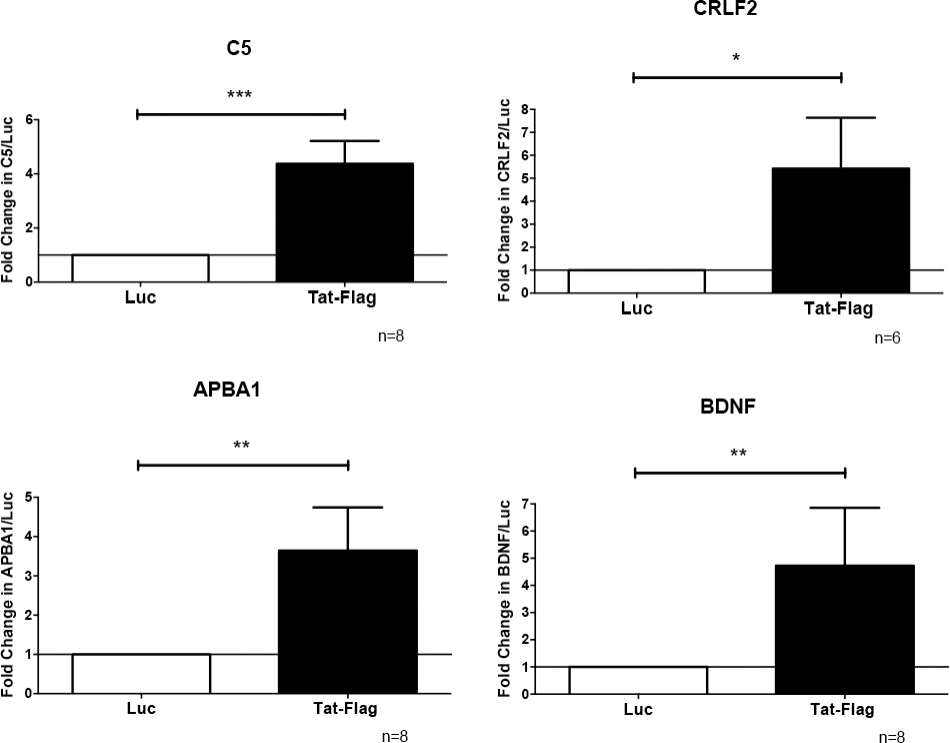
Tat complex binds to the human macrophage genome at C5, CRLF2, APBA1, and BDNF. THP-1-Tat-Flag and THP-1-luciferase cell lines generated from 5–6 independent sets of infection and selection were treated with PMA for 24 hours, cell lysates were prepared, and immunoprecipitated with antibody to Flag, followed by qPCR analysis. For qPCR analysis, we used the specific sequences in the genome identified by ChIP-seq and designed specific primers for these regions for the 4 genes (C5, CRLF2, APBA1, and BDNF). The precipitated DNA and input were then quantified by real-time PCR. Quantification is expressed in arbitrary units indicating fold over control (Luc), and target sequence levels were normalized to the input using the method of Pfaffl. Tat complex significantly bound to the 4 human macrophages genes selected, C5, CRLF2, APBA1, and BDNF. Significance was determined using a Wilcoxon signed-rank test. Error bars represent the median ± S.D.; *P < 0.05, **P < 0.01.

**Fig 4:**
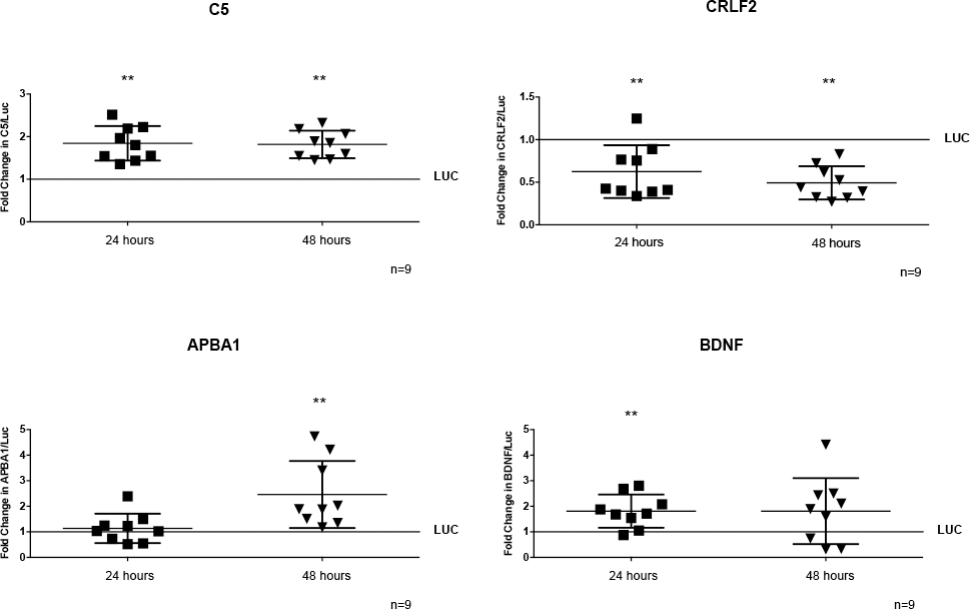
Quantitative RT-qPCR demonstrate that Tat complex modulates expression of C5, CRLF2, APBA1, and BDNF. THP-1-Tat-Flag and THP-1-luciferase cell lines generated from 5–6 independents sets of infection and selection were differentiated to macrophages with PMA for 24 and 48 hours. Total RNA was extracted, and cDNA was generated and a RT-qPCR performed. Quantification is expressed in arbitrary units, relative to THP-1-luciferase set to 1, and target mRNA levels were normalized to 18S expression using the method of Pfaffl. Tat complex increased the expression of C5 at 24 and 48 hours, decreased the expression of CRLF2 at 24 and 48 hours, increased the expression of APBA1 at 48 hours and increased the expression of BDNF after 24 hours of PMA treatment. Significance was determined using a Wilcoxon signed-rank test. Error bars represent the median ± S.D.; **P < 0.01.

We confirmed the ChIP-seq results by 2 different methods. First, we examined the association of the Tat complex with genes/promFoters by ChIP assay. We used the sequences in the genome identified by ChIP-seq and designed specific primers for these regions, using the online software Primer4 (http://frodo.wi.mit.edu/) and Primer-Blast (http://www.ncbi.nlm.nih.gov/tools/primer-blast/) (S1 Table). We performed an additional 4–5 independent sets of infections and selection of THP-1-Tat-Flag and THP-1-luciferase cell lines. These were included in our experiments as well as the original cell lines described in Figs 1 and 2. Cell lines were treated with PMA for 24 hours to induce their differentiation to macrophages, cell lysates were prepared and immunoprecipitated with antibody to Flag, followed by qPCR analysis. As shown in Fig 3, the association of Tat-Flag complex is specific to all the regions analyzed (C5, CRLF2, APBA1, and BDNF). Second, we analyzed the expression of the genes identified by ChIP-seq in THP-1-Tat-Flag and THP-1-luc cells treated with PMA for 24 and 48 hours by qRT-PCR. We studied two time points (24 and 48 hours) because the kinetics of gene expression are not similar for all genes, and we were uncertain which genes would be expressed at a given time point in our experiments. Also, we examined CD14 on these cells (0, 24 and 48 hours after PMA treatment), and did not detect any significant differences at any time point or that correlated with changes in expression of the newly characterized genes (data not show). Thus, the differences in gene expression cannot be ascribed to the maturation state of the cells Tat complex increased the expression of C5 at both time points, decreased the expression of CRLF2 at 24 and 48 hours after PMA treatment, increased the expression of APBA1 after 48 hours of treatment with PMA, and increased the expression of BDNF after 24 hours of PMA treatment (Fig 4).

These data demonstrate that Tat complex binds to this human macrophage genome and modulates expression of C5, CRLF2, APBA1, and BDNF.

### In silico analysis of transcription factors for targets to which Tat may bind and regulate host gene expression

To characterize one of the mechanisms by which Tat complex modulates gene expression in human macrophages, we analyzed the sequence of four promoters of the genes found by ChIP-seq (C5, CRLF2, APBA1, and BDNF) for potential transcription factor binding sites, using Multisearch sites ALGGEN-PROMO software, with a dissimilarity of 5% [66, 67]. We found several common transcription factors that potentially interact with the promoters analyzed (S2 Table). Among these transcription factors, Tat has been found to interact directly with p53 [35], C/EBP [36], ETS1 [37], IRF-1 [68], and TFIID [69]. Using this *in silico* analysis, we propose a possible mechanism of regulation of gene expression that involves the association of Tat with one or more transcription factors to form a Tat complex that regulates gene expression.

### Lysine 50 of Tat regulates gene expression in differentiated THP-1 cells expressing Tat-Flag

**Fig 5:**
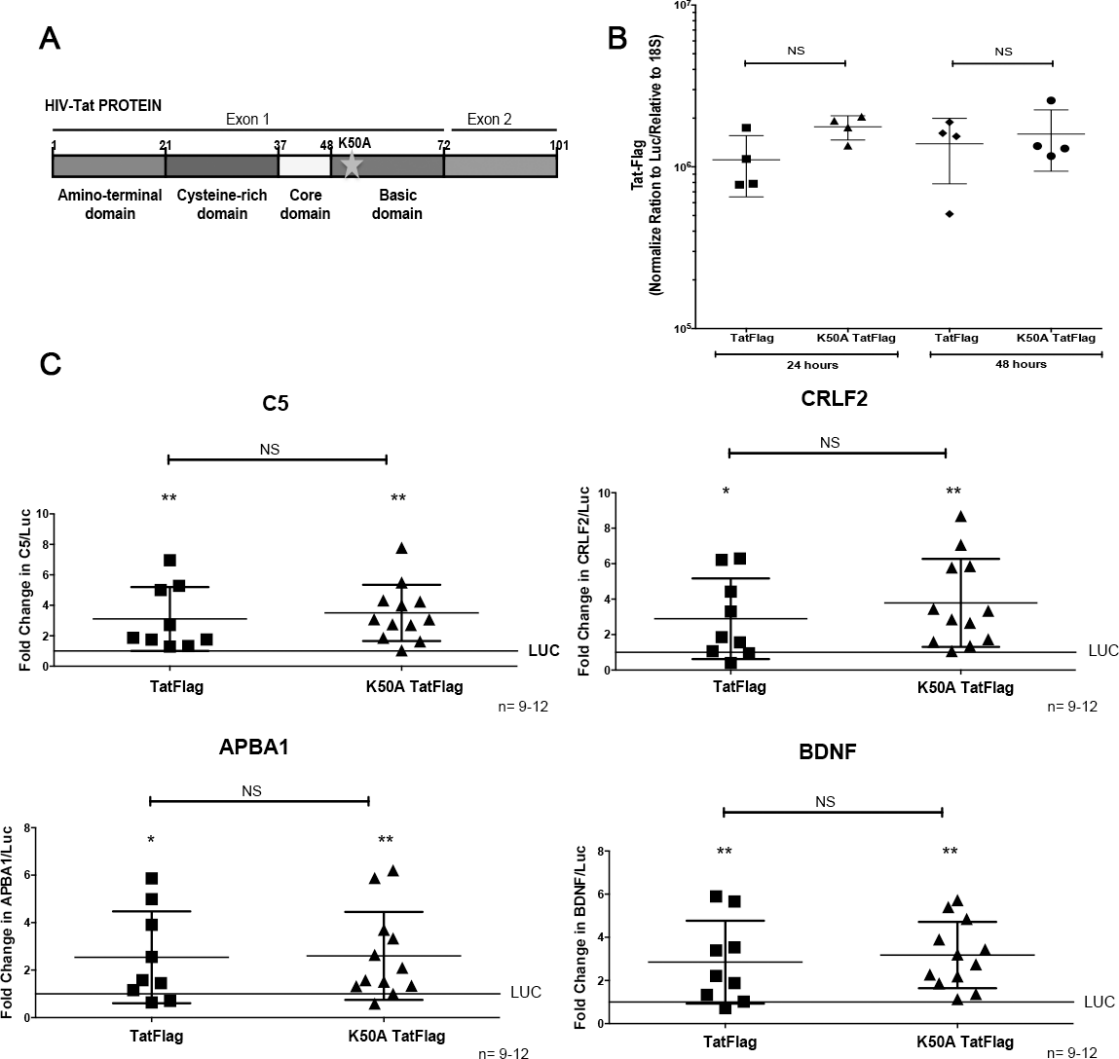
Tat-Flag and mutant K50ATat-Flag complex bind similarly to human C5, CRLF2, APBA1, and BDNF genes. **(A)** Cartoon of HIV-Tat protein illustrating the mutation K50A. We infected THP-1 cells with a lentivirus with this mutation to generate stable cells that express K50ATat-Flag. **(B)** Expression of Tat-Flag and K50ATat-Flag at 24 and 48 hours was analyzed by qRT-PCR. THP-1Tat-Flag cell lines generated from 5–6 independent sets of infection and selection, and THP-1TatK50ATat-Flag cell lines generated from 2 independent sets of infection and selection, were differentiated to macrophages with PMA for 24 and 48 hours. Total RNA was extracted and cDNA was generated. The target mRNA levels were normalized to 18S using the method of Pfaffl. We found similar expression of Tat-Flag and K50ATat-Flag genes. **(C)** THP-1Tat-Flag and THP-1Tat K50ATat-Flag cell lines were treated with PMA for 24 hours, cell lysates were prepared and immunoprecipitated with antibody to Flag, followed by qPCR analysis. For qPCR analysis, we used the specific sequences in the genome identified by ChIP-seq and we designed specific primers for these regions for the 4 genes, C5, CRLF2, APBA1, and BDNF. The precipitated DNA and input were quantified by real-time PCR. Quantification is expressed in arbitrary units indicating fold change over control (Luc), and target sequence levels were normalized to the input using the method of Pfaffl. No significant differences were found between the association of Tat-Flag and K50ATat-Flag to the 4 genes selected. Significance was determined using a Wilcoxon signed-rank test. Error bars represent the median ± S.D.; *P < 0.05, **P < 0.01. For comparison between Tat-Flag and K50ATat-Flag gene expression, Mann-Whitney tests were performed. Error bars represent the median ± S.D., ns: not significant.

**Fig 6:**
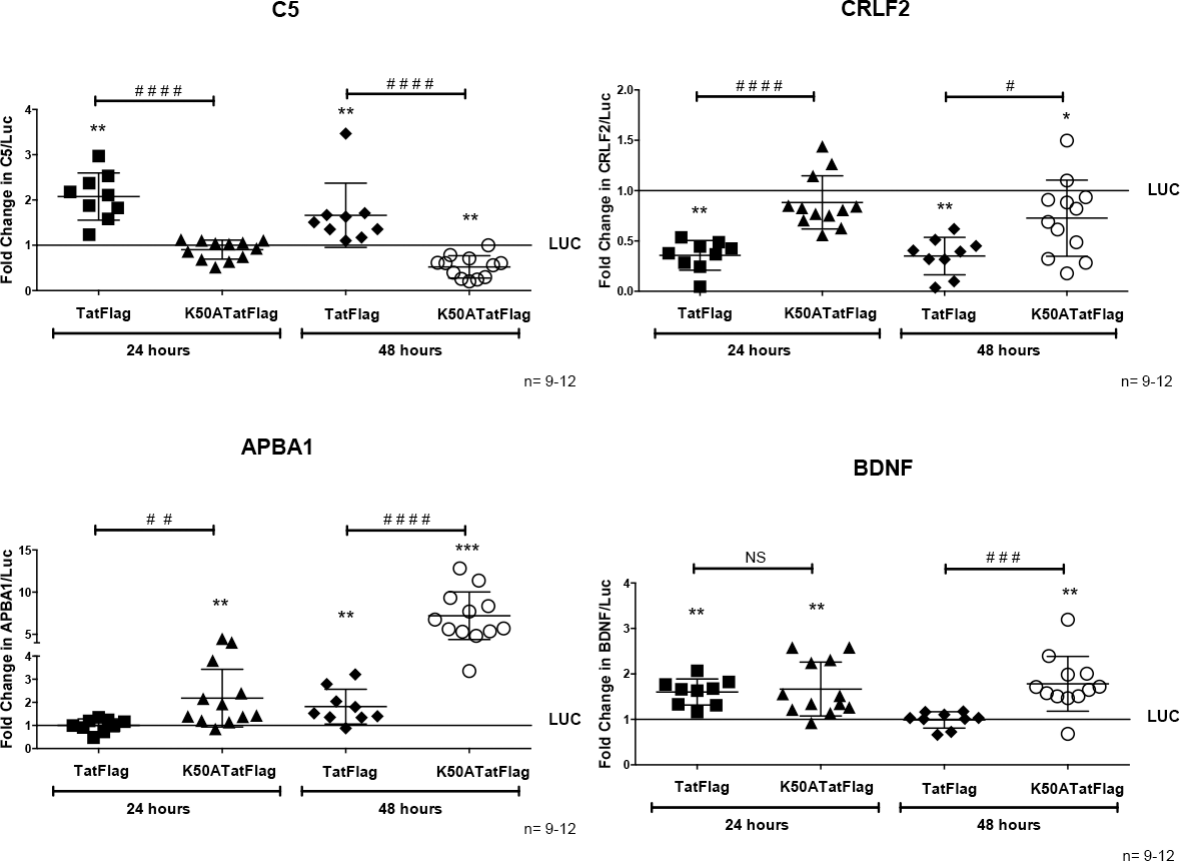
Lysine 50 of Tat regulates expression of C5, CRLF2, APBA1, and BDNF genes. THP-1Tat-Flag and THP-1Tat K50ATat-Flag cell lines (generated from independent experiments described in Fig 5) were differentiated to macrophages with PMA for 24 and 48 hours. Total RNA was extracted, and cDNA was generated. Quantification is expressed in arbitrary units, relative to THP-1-luciferase set to 1, and target mRNA levels were normalized to 18S expression using the method of Pfaffl. The K50ATat-Flag mutant decreased Tat-Flag complex mediated C5 expression, and its expression is similar to the control at 24 hours. For CRLF2, this mutant had a similar effect on gene expression as the control. K50ATat-Flag mediated a very significant increase in gene expression of APBA1 at both time points in comparison to wild type Tat-Flag complex and the control. For BDNF, K50ATat-Flag had the same effect on gene expression as did wild type Tat-Flag at 24 hours, and increased BDNF expression at 48 hours, in comparison to Tat-Flag and the control. Significance (*) between control (Luc) and Tat-Flag was determined using a Wilcoxon signed-rank. Error represents the median ± S.D.; *P < 0.05, **P < 0.01, ***P < 0.001. For comparison between Tat-Flag and K50ATat-Flag (#) gene expression, Mann-Whitney tests were performed. Error represents the median ± S.D.; ^#^P < 0.05, ^##^P < 0.01, ^###^P < 0.001, ^####^P < 0.0001, NS: not significant.

The lysine 50 of Tat, in its basic domain, may play an important role in gene regulation. It can be acetylated by p300 [70, 71], and can be methylated by SETDB1/SETDB2 [72], which have been shown to regulate Tat’s transcriptional activity. We examined the role of lysine 50 of Tat in the regulation of the genes C5, CRLF2, APBA1, and BDNF by changing the Lysine 50 to alanine (K50ATat-Flag), and generated stable THP-1 mutant cell lines that express K50ATat-Flag (Fig 5A). We examined the expression of Tat-Flag and K50ATat-Flag at 24 and 48 hours by qRT-PCR and we found no significant differences in the expression of both genes (Fig 5B). Thus, the mutation did not change the expression of Tat in the THP-1 cells. Next, we studied the association of Tat-Flag-complex (Tat wild type and K50ATat) with the genes C5, CRFL2, APBA1 and BDNF by ChIP assay, and analyzed the contribution of lysine 50 of Tat to their expression by qRT-PCR. We found association of both the wild type Tat-Flag complex and K50ATat-Flag complex to all the regions analyzed (C5, CRLF2, APBA1, and BDNF), with no significant differences between Tat-Flag and K50ATat-Flag complex, suggesting that the mutant K50ATat-Flag does not affect the association of the complex with the genes analyzed (Fig 5C). Nonetheless, we found that K50ATat-Flag regulates expression of these genes. Wild type Tat-Flag complex increased the expression of C5, decreased the expression of CRLF2, increased the expression of APBA1, and increased the expression of BDNF, as shown in Fig 4. However, the K50ATat-Flag mutant decreased the C5-induced expression by Tat-Flag complex (Fig 6). For CRLF2, this mutant does not have an effect on gene expression. For APBA1, K50ATat-Flag mediated a very significant increase in gene expression at all time points in comparison to wild type Tat-Flag complex and the control. For BDNF, K50ATat-Flag had the same effect on gene expression as did wild type Tat-Flag at 24 hours, and increased BDNF expression at 48 hours, in comparison to Tat-Flag and the control (Fig 6). All of these data suggest that lysine 50 of Tat is regulating the expression of C5, CRLF2, APBA1, and BDNF.

## Discussion

HIV infected people are living much longer. However, with the success of antiretroviral therapy, 40–70% of the HIV-infected population exhibits HAND, characterized by cognitive deficits, motor abnormalities, and behavioral changes [3, 18, 73]. This occurs despite cART, and is believed to be initiated and mediated by CNS HIV infection and the establishment of CNS viral reservoirs.

HIV-infected monocytes bring virus into the CNS where they can differentiate into macrophages, resulting in HIV infection of the brain, and in the activation of CNS cells incluiding macrophages, microglia, and astrocytes. These cells produce cytokines, chemokines, and viral proteins that promote neuroinflammation and neuronal damage [19, 74–76]. HIV-infection of macrophages does not cause cell death, resulting in the establishment of long-lived macrophage viral reservoirs [17, 22, 47, 77–79]. Importantly, Tat is still produced by infected macrophages, even in the context of antiretrovirals and suppression or minimal viral replication [23, 46, 80].

Tat is the transactivator of transcription that is essential for transcriptional regulation of HIV. Tat does not have any DNA binding domains. Studies have shown that Tat binds to many proteins (transcription factors, activators, and repressors of transcription), and that the Tat complex can regulate host gene expression. For example, Tat can interact with RNA polymerase II, General Transcription Factors (TFIIB, TFIID, TFIIH, and TAF55), transcription regulators p300/cAMP response element-binding protein (CREB), p300/CBP-associated factor (p/CAF), Tip60, human Nucleosome Assembly Protein-1 (hNAP-1), as well as with the nucleosome remodeling complex SWI/SNF, and transcription factors (Sp1, NFAT, AP-1, NF-kB complex, p53, C/EBPb) [26–36].

In this *in vitro* study, we infected the monocyte/macrophage THP-1 cell line with a lentivirus to express Tat-Flag. Thus, we are examining the impact of one viral protein (Tat) on host gene regulation. The advantage of this approach is that it enables us to isolate the effects of Tat by itself, without other cellular or viral contributions. In the presence of ART, Tat is still produced so it would still play an important role in HIV neuropathogenesis. While studying the effects of tat by itself is a strength, the limitations of this study are that other factors will be important *in vivo* that are not being addressed in our experiments.

We demonstrated that Tat protein regulates gene expression in macrophages using ChIP-seq analyses. We identified 66 binding sites of Tat associated with macrophage genes, and grouped them using IPA to identify the networks in which they participate. Among the genes, there are some that were shown to have a role in inflammation and chemotaxis, including C5, and CRLF2/TSLPR, and others that have a role in neuronal function such as APBA1/Mint, and BDNF. We chose to focus our first set of analyses on these four genes.

C5 is part of the complement system, and activation of complement leads to cleavage of C5, resulting in the generation of C5a and C5b. C5a is a potent inflammatory mediator [81, 82]. C5 is synthesized by macrophages [83–86], and HIV infection increases the expression of the complement system in CNS cells [87–90]. HIV-mediated C5a production induces chemotactic responses of monocytes, macrophages, and dendritic cells [58, 91, 92]. In human brain microvascular endothelial cells, Tat increases the expression of C3 and C7 [93]. In the CSF of individuals with HIV, levels of C3 and C4 are increased compared with those of uninfected people [94], and in brain tissues of infected individuals with HIV-1, C3 was significantly higher as compared to uninfected brain [95]. We found that Tat increases the expression of another component of complement system, C5. Thus, we propose that HIV Tat from infected macrophages, even in the absence of active viral replication, may induce C5 expression that would contribute to neuroinflammation.

CRLF2/TSLPR, is a member of the cytokine type I receptor family that forms a functional heterodimeric complex with the IL-7 receptor [96, 97]. Many immune cells express both of these receptors, including monocytes/macrophages [96–98]. CRLF2/IL7R signaling is involved in the development of the hematopoietic system, cell proliferation, survival, and inflammatory responses [60, 61, 97–99]. The role of HIV or HIV Tat in the expression of CRLF2 in macrophages is unknown. The expression of the IL-7 receptor is decreased in T cells from HIV-infected people [100, 101]. HIV Tat also downregulated IL-7 receptor in T cells [102]. In HBMEC, HIV Tat induced upregulation of CRLF2 as shown by microarray [93]. CRLF1, another cytokine type I receptor family member, is downregulated by HIV-1 Tat in macrophages [39]. We found CRLF2/TSLPR is decreased in the cell lines that express Tat-Flag. Thus, CRLF2/IL7R signaling is involved in the inflammatory response and HIV Tat may downregulate CRLF2, which suggests that HIV infection would result in increased inflammatory responses regulate by this gene.

Another gene identified in our study is APBA1/Mint1, a member of the X11 protein family, a neuronal adaptor protein that interacts with beta-amyloid precursor protein (APP) [103, 104] and modulates the processing of amyloid precursor protein and amyloid beta production. There are conflicting data about the functions of APBA1. Some groups showed that overexpression of APBA1/Mint decreases amyloid beta [103, 105, 106]. Alternatively, experiments that deleted APBA1/Mint in mice showed that amyloid production decreased [107, 108]. APBA1/Mint1 was upregulated in the brain tissue of people with Alzheimer’s disease [109]. Beta-amyloid precursor protein (APP) and amyloid beta were found in macrophages in brain sections from individuals with HIV, and HIV Tat increased amyloid beta in neuron cultures [110–113]. We found increased expression of APBA1/Mint1 in the cell lines that express Tat-Flag, which suggests that HIV-1 Tat may contribute to neurodegeneration in the pathogenesis of HAND by modulating the processing of APP, and thereby increasing amyloid beta production.

BDNF is a neurotrophin that promotes neuron survival. Mature BDNF is generated after proteolytic cleavage of ProBDNF and is released from macrophages during inflammation and injury [114]. ProBDNF induces neuronal apoptosis and BDNF increases survival of neurons [115, 116]. Neuronal survival or death depends on the ratio of proBDNF/BDNF. HIV alters the processing of proBDNF, and in the CSF or brains of HIV infected people with HIV encephalitis, there was a decrease in BDNF and an increase in proBDNF [117–119]. Two HIV proteins contribute to the decreased processing of proBDNF to BDNF in neurons, Tat [63] and gp120 [117]. We propose that this Tat-mediated dysregulation in proBDNF/BDNF could contribute to neuronal damage in the pathogenesis of HAND.

The transcriptional activity of Tat is also regulated by post-translational modifications. Tat modifications can be activating or inhibitory. Lysine 50 of Tat plays an important role, as it can be acetylated by p300 [70, 71], or it can be methylated by SETDB1/SETDB2 [72]. Acetylated Tat 50 facilitates the interaction of Tat with the SWI/SNF chromatin-remodeling complex and with other histone acetyltransferase PCAF [120–125]. This acetylation can be deacetylated by the class III HDAC or also named SIRT1 [126]. Deacetylation of Tat by SIRT1 could play a significant role in Tat’s transcriptional activity, in that it would enable it to undergo a new transcription cycle [125, 126]. Methylation of lysine 50 of Tat would indicate the recruitment of corepressor complexes and remodelling chromatin for repression and thereby, initiatiation of transcriptional silencing [72]. Additionally, these post-translational modifications in Tat may contribute to viral latency [125] in HIV infected cells.

The acetylation or methylation of lysine 50 of Tat may affect the expression of target genes of the Tat complex. In this study, we showed a potential mechanism by which lysine 50 of Tat regulates gene expression in human macrophages. The mutation of lysine 50 to alanine did not affect the association of the Tat-Flag-complex to the regions analyzed (C5, CRLF2, APBA1, and BDNF), as compared with the association of the wild type Tat-Flag complex. However, K50ATat-Flag complex regulated gene expression. K50ATat-Flag decreased C5 as compared with the wild type Tat-Flag complex. K50ATat-Flag has a significant increase on APBA1 during all time points. For BDNF, K50ATat-Flag had the same effect on gene expression as did Tat-Flag wild type at 24 hours, and increased BDNF expression above wild type Tat-Flag complex at 48 hours. However, this mutant did not affect the expression of CRLF2 in comparison with the control. All these data suggest that lysine 50 of Tat is regulating the expression of C5, CRLF2, APBA1, and BDNF. The lysines at position 50 and 51 in Tat are important for RNA binding activity. Tat interacts with multiple host cellular mRNAs in human T cells and this interaction depends on Lysine 50 and 51. That interaction could regulate the mRNA transcription, processing, export or stability [127]. Future studies will examine the mechanisms by which lysine 50 of Tat dysregulates this gene expression to characterize how macrophage reservoirs in the brain contribute to neuroinflammation and CNS damage in the pathogenesis of HAND.

With the success of cART, HIV infected people live longer, and HIV-associated neurocognitive disorders still remain as a major public health issue. HIV Tat is produced by HIV infected cells despite cART treatment, even when HIV replication is minimal or suppressed, contributing to chronic neuroinflammation and neuronal damage. CNS HIV reservoirs, comprised of infected macrophages, persist for very long periods of time and are one of the main obstacles to HIV eradication. The dysregulation of host genes by HIV Tat in human macrophages found in this study would contribute to this low level of CNS inflammation and neuronal damage. Lysine 50 of Tat could be a potential therapeutic target to prevent or decrease the neurological damage in the brain of HIV infected people, and together with the current antiretrovirals, provide new therapeutic benefits.

## Supporting information

S1 Table qPCR Primers used for ChIP assay. (PDF)

S2 Table Potential common transcription factor binding sites using Multisearch sites ALGGEN-PROMO software, in C5, CRLF2, APBA1 and BDNF genes. (PDF)
