# Supplementary material for: HIV-Tat regulates macrophage gene expression in the context of neuroAIDS
Source: PLoS One. 2017 Jun 22;12(6):e0179882. doi: 10.1371/journal.pone.0179882 (PMC5481010; doi:10.1371/journal.pone.0179882)
Supplement: S1 Table — (PDF) [file pone.0179882.s001.pdf]

Supplementary Table 1. qPCR Primers used for ChIP assay

Primer	Sequence 5'-3'
C5 Forward	AGGGGACATGACCAGACAAC
C5 Reverse	TGAGGACAGCAGATCATCCA
CRLF2 Forward	CCTCAGGGACCTACAACGAA
CRLF2 Reverse	CACTGTGCTTCCTGCCTGTA
APBA1 Forward	ACTGATTTGAGGCCATCCTG
APBA1 Reverse	CTTGTGTGCCCTTGGGTAGT
BDNF Forward	GTCTCGCTCCCCTAGCTTTC
BDNF Reverse	AAGCTCCGTAGTGCAGGAAG
Tat-Flag Forward	AGCGACGAAGACCTCCTCAA
Tat-Flag Reverse	TCATCGTCGTCCTTGTAGTCG
