# Supplementary material for: HIV-Tat regulates macrophage gene expression in the context of neuroAIDS
Source: PLoS One. 2017 Jun 22;12(6):e0179882. doi: 10.1371/journal.pone.0179882 (PMC5481010; doi:10.1371/journal.pone.0179882)
Supplement: S2 Table — (PDF) [file pone.0179882.s002.pdf]

Supplementary Table 2. Potential common transcription factor binding sites using Multisearch sites ALGGEN-PROMO software, in C5, CRLF2, APBA1 and BDNF.

Transcription Factors	Genes: C5	CRLF2	APBA1	BDNF
GATA-1	x	x	x	
GR-beta	x	x	x	x
C/EBPbeta	x	x	x	x
TFIID	x	x		x
FOXP3	x	x		x
TFII-I	x	x	x	x
STAT4	x	x	x	x
c-Ets-1	x	x	x	x
YY1	x	x	x	x
AP-2alphaA	x	x	x	x
Pax-5	x		x	x
p53	x		x	x
GR-alpha	x	x	x	x
IRF-1	x	x	x	x
RXR-alpha	x	x	x	x
c-Ets-2	x	x	x	x
MAZ		x	x	x
